# The effectiveness of an integrated CBL and BOPPPS model in neurological nursing clinical teaching: a comparative study

**DOI:** 10.3389/fmed.2026.1789025

**Published:** 2026-07-29

**Authors:** Peijun Qian, Juan Su, Yaoyao Xu

**Affiliations:** Department of Neurology, The Second Affiliated Hospital of Anhui Medical University, Hefei City, Anhui Province, China

**Keywords:** BOPPPS, CBL, clinical practice teaching, neurological nursing, nursing education, quasi-experimental study

## Abstract

**Objective:**

This study aimed to develop an integrated teaching plan that combines case-based learning (CBL) with the BOPPPS teaching model, its application effect in clinical practice education for neurological nursing, and provide an optimized pathway for training specialized nursing personnel.

**Methods:**

We conducted a single-centre, non-randomized quasi-experimental study with two parallel groups. Eighty-six nursing students who completed internships in our hospital from September 2024 to August 2025 were allocated to two groups by internship batch (43 per group); allocation was therefore non-random. The control group received the lecture-based learning (LBL) teaching model, and the experimental group received the combination of CBL and BOPPPS teaching model. Teaching effectiveness was compared between groups using theoretical assessments, the Objective Structured Clinical Examination (OSCE), the Chinese Version of the Critical Thinking Disposition Inventory (CTDI-CV), and a satisfaction survey. Between-group differences were analysed with independent-samples *t*-tests, corroborated by Mann–Whitney U tests, and reported with Cohen’s d effect sizes.

**Results:**

The experimental group scored significantly higher than the control group in theoretical knowledge (87.62 vs. 78.45; Cohen’s d = 1.51). Clinical practical ability, measured by OSCE, was also significantly higher in the experimental group (85.36 vs. 76.28; d = 1.72). The experimental group also achieved a higher post-internship CTDI-CV total score (289.57 vs. 269.63; d = 1.01). Teaching satisfaction was higher in the experimental group (mean overall score of 92.45 vs. 81.36; d = 2.44).

**Conclusion:**

In this quasi-experimental setting, the integrated CBL and BOPPPS model was associated with improved theoretical knowledge, clinical practical ability, and critical thinking dispositions of nursing interns in neurology, and with higher students’ learning satisfaction. These findings suggest the model is a promising approach to clinical teaching that warrants further multi-centre, randomized evaluation before broad promotion in specialized nursing education.

## 1 Introduction

Neurological diseases are characterized by complex etiology, variable progression, and diverse clinical manifestations, encompassing conditions such as stroke, epilepsy, and Parkinson’s disease. Meanwhile, these disorders are often accompanied by various symptoms including dizziness, headache, limb numbness and weakness, disturbance of consciousness, and motor dysfunction (1–3). These diseases entail not only clinical complexities but also the necessity for nursing care that is both comprehensive and patient-centered, encompassing support for the psychological and emotional well-being of patients and families (4). Therefore, the professional knowledge reserve, clinical judgment ability, and emergency response capacity of nursing staff directly determine the outcomes of nursing work (5).

The quality of clinical practice education, which acts as a vital bridge between nursing theory and clinical practice, has a profound impact on the effectiveness of training healthcare personnel (6). The traditional educational model, primarily consisting of lecture-based learning (LBL) and demonstrations with students in a passive listening role, has been widely used in China (7, 8). Due to its singular methodology, lack of interactivity, and limited practicality, this model fails to fully engage students’ initiative and restricts their comprehensive development, often resulting in suboptimal teaching quality, academic performance, and student evaluations (9).

Although case-based learning (CBL) and the BOPPPS framework have each been evaluated in health-professions education, few studies have examined their structured integration in the highly specialized context of neurological nursing, where rapid clinical reasoning and emergency response are essential. This gap motivated the present study.

Accordingly, this study addressed three research questions (RQs): RQ1—Is an integrated CBL–BOPPPS model associated with greater gains in theoretical knowledge and OSCE-assessed clinical skills than traditional LBL among neurological nursing interns? RQ2—Is the integrated model associated with improved critical thinking disposition (CTDI-CV), and which sub-dimensions respond most? RQ3—Do learners report higher satisfaction with the integrated model? The principal contribution of this work is the first quasi-experimental evaluation of a phase-by-phase CBL–BOPPPS integration tailored to neurological nursing, accompanied by effect-size reporting and a transparent appraisal of its non-randomized design.

## 2 Literature review

This section synthesizes prior work on the two component pedagogies and the rationale for their integration, and articulates the research gap addressed here.

### 2.1 Case-based learning (CBL)

By engaging students in the analysis, discussion, and reflection of authentic clinical cases, Case-Based Learning (CBL) effectively stimulates learning initiative. This process not only promotes deep integration of theoretical knowledge with clinical practice but also cultivates essential clinical thinking and problem-solving abilities (10). Recent studies have confirmed that this model can enhance the learning experience while equipping students with the competencies required for clinical practice (11). A systematic review and meta-analysis further reported that CBL improves critical thinking dispositions among Chinese nursing students, although effect sizes varied with implementation fidelity and case quality (12). Nevertheless, CBL used in isolation may lack an explicit structure for goal-setting, diagnostic pre-assessment, and closed-loop feedback, which can limit the consistency of learning gains (11).

### 2.2 The BOPPPS model

The BOPPPS teaching model, also known as the guided learning interactive efficiency-enhancing teaching method, was initially developed to improve teaching effectiveness and instructor skills for teacher training (13). This model is a closed-loop instructional design framework based on constructivist theory (14), dividing classroom teaching into six modules: Bridge-in, Objective, Pre-assessment, Participatory Learning, Post-assessment, and Summary. The Bridge-in phase introduces the lesson through problem-oriented scenarios to capture student attention and stimulate interest. Objectives specify the learning goals for students, which are designed to be both measurable and attainable according to their respective levels. Pre-assessment is the process of gauging students’ existing knowledge through diagnostic evaluation before class, thereby informing instructors’ adjustments to instructional content and pace. Participatory Learning is characterized by a dynamic process of interaction and collaboration between teachers and students, designed to achieve specific learning objectives. Post-assessment is the process of measuring student learning outcomes against the intended objectives at the conclusion of a teaching session, thereby evaluating the effectiveness of the instruction. Summary involves consolidating key content and core concepts (15, 16).

Grounded in a student-centered philosophy, the BOPPPS model orchestrates a systematic learning process by effectively connecting the six stages of its instructional modules. Evidence suggests that this student-centered model not only improves teaching efficiency but also offers marked advantages over traditional instruction in terms of course satisfaction, teacher-student interaction, and the enhancement of clinical analytical abilities (7, 32). However, because BOPPPS chiefly governs the sequence and structure of a session, it does not by itself supply the rich, authentic clinical content that drives contextual reasoning (17).

### 2.3 Integrated CBL–BOPPPS approaches and research gap

The two models are theoretically complementary: BOPPPS provides the structural scaffold (clear objectives, diagnostic pre-assessment, participatory activity, and closed-loop post-assessment and feedback), while CBL supplies authentic clinical cases that anchor each phase in concrete reasoning. Embedding a single longitudinal case across all six BOPPPS phases therefore couples structure with context, so that pre-assessment targets case-relevant gaps, participatory learning operationalizes case analysis, and post-assessment verifies case-based decisions. Integrated CBL–BOPPPS designs have shown favourable results in ophthalmology, oral and maxillofacial surgery, and general nursing education (7, 18, 19). However, evidence in neurological nursing—where time-critical decision-making is paramount—remains scarce, and prior reports seldom quantify effect sizes or address the limitations of non-randomized allocation. The present study addresses this gap.

## 3 Materials and methods

### 3.1 Study design

This was a single-centre, two-group, non-randomized quasi-experimental study with a pre-test/post-test design. Participants were allocated to the experimental or control group by internship batch rather than by randomization; consequently, no allocation sequence, concealment, or randomization procedure was used, and the design does not constitute a randomized controlled trial. The study was reported in accordance with the TREND statement for non-randomized evaluations (completed checklist provided as Supplementary material). The study protocol received review and approval from the Institutional Review Board (IRB) of the Second Affiliated Hospital of Anhui Medical University (approval no. 2024-LW-035), and written informed consent was obtained from all participants. The research was conducted in compliance with the Declaration of Helsinki.

### 3.2 Participants

A convenience sample of eighty-six nursing students who completed their clinical practice in the hospital’s Department of Neurology between September 2024 and August 2025 were enrolled.

*Inclusion criteria*: (1) Full-time undergraduate or college nursing students; (2) Entering clinical internship in neurology with a 4-week internship cycle; (3) Voluntary participation in the study with signed informed consent.

*Exclusion criteria*: (1) Internship interruption exceeding 3 cumulative days during the study period due to special reasons; (2) History of neurological diseases or cognitive dysfunction; (3) Refusal to participate in the study or failure to complete all evaluation indicators.

**Figure 1:**
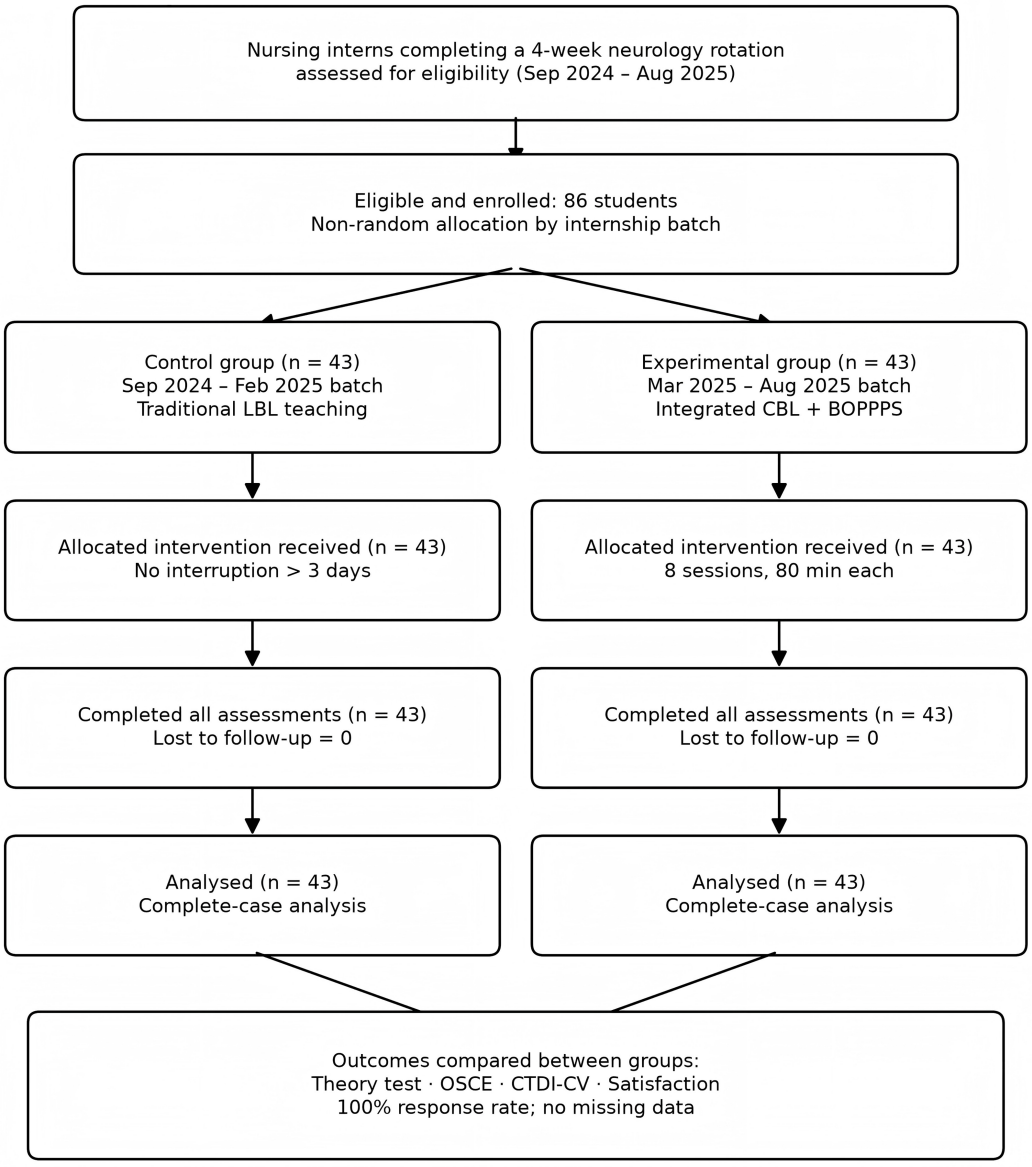
CONSORT flow diagram of study participants. LBL, lecture-based learning; CBL, case-based learning; BOPPPS, bridge, objectives, pre-assessment, participation, post-assessment, summary; OSCE, objective structured clinical examination; CTDI-CV, california critical thinking disposition inventory-chinese version.

*Participants were grouped by internship batch*: 43 students interning from September 2024 to February 2025 comprised the control group, and 43 students interning from March to August 2025 comprised the experimental group. Participant flow is summarized in Figure 1. The control group included 5 males and 38 females; aged 20–23 years, mean (21.56 ± 0.87) years; educational background: 22 undergraduates, 21 college students; pre-internship basic nursing theory score (79.32 ± 6.45). The experimental group included 4 males and 39 females; aged 20–24 years, mean (21.78 ± 0.92) years; educational background: 23 undergraduates, 20 college students; pre-internship basic nursing theory score (78.95 ± 6.73). No statistically significant differences were found between the two groups in general characteristics such as gender, age, educational background, or pre-internship basic nursing theory scores (*p* > 0.05), indicating comparability.

*Sample-size justification*: the study size was constrained by the available internship cohorts during the study window. An *a priori* calculation indicated that, to detect a moderate-to-large between-group difference (Cohen’s d = 0.70) in the primary theoretical-knowledge outcome with two-sided *α* = 0.05 and 80% power using an independent-samples t-test, approximately 34 participants per group were required. The enrolled 43 per group (86 total) therefore provided adequate power (>0.80) to detect effects of d ≥ 0.62, and the observed primary effects (d ≥ 1.5) were considerably larger. The study was nonetheless not powered to detect small subscale differences, which is acknowledged as a limitation.

### 3.3 Intervention protocol

#### 3.3.1 Control group

The traditional clinical teaching model for neurological nursing was applied, including the following specific contents: (1) Ward rounds with preceptors: Following instructors during daily ward rounds to observe patient condition changes and nursing procedures; (2) Theoretical lectures: One centralized weekly lecture using PPT, covering etiology, clinical manifestations, treatment principles, and nursing key points of common neurological diseases; (3) Skill demonstration: Instructors demonstrating common neurological nursing procedures, followed by student practice under instructor guidance; (4) Case explanation: Instructors explaining clinical cases, with students passively receiving knowledge without dedicated CBL sessions. The internship cycle was 4 weeks.

#### 3.3.2 Experimental group

Based on the traditional teaching of the control group, the integrated CBL and BOPPPS teaching model was implemented as follows:

*Faculty preparation*: three clinical nursing instructors with over 5 years of neurological nursing experience, bachelor’s degrees or higher, holding positions of charge nurse or above, and possessing rich clinical teaching experience were selected. They received specialized training in the BOPPPS teaching model and CBL facilitation to ensure standardized implementation.

*Teaching cycle and frequency*: a 4-week internship cycle with 2 integrated teaching sessions per week, each lasting for 80 min, totaling 8 sessions.

*Case selection*: eight typical neurological cases were selected, including acute ischemic stroke (3 cases), epilepsy (1 case), Parkinson’s disease (1 case), multiple sclerosis (1 case), Guillain-Barre syndrome (1 case), and vascular dementia (1 case). All cases were derived from real, anonymized patients with complete data (history, physical examination, auxiliary tests, condition changes, treatment plans) and clear teaching focus and discussion value. Cases were chosen by consensus of three senior neurology nurses to span the core competencies of the internship curriculum and the most frequent and highest-acuity presentations on the ward (ischaemic stroke being the most common admission), ensuring curricular alignment and clinical representativeness (18, 20).

*Specific teaching implementation*: strictly followed the integrated teaching framework: Bridge-in: Presenting typical clinical symptoms or urgent scenarios from the case to provoke student thinking. Objective Clarification: Defining knowledge objectives, ability objectives, and quality objectives. Pre-assessment: Using 3–5 short-answer or multiple-choice questions to assess students’ foundational knowledge. Participatory Learning: Students divided into groups of 5–6, each electing a leader. Groups discussed the case, analyzing nursing problems, potential risks, nursing measures, and formulated detailed nursing plans. Role-playing simulated patient care processes. Instructors circulated among groups, providing guidance, correcting misconceptions, and answering questions. Post-assessment: Providing follow-up treatment and condition change data for the case, asking students to analyze nursing effectiveness and adjust care plans. Scenario simulations assessed mastery of core nursing skills. Summary: Instructors summarized core nursing points and clinical reasoning pathways for the case, highlighted strengths and weaknesses in group discussions and role-plays, reinforced key knowledge, and addressed learning objectives.

Intervention fidelity: to maintain consistency across the 8-month period and across the three instructors, all sessions followed a standardized lesson plan and a structured BOPPPS facilitation checklist specifying the activities and time allocation for each of the six phases. Instructors attended a calibration workshop before the study and a brief monthly refresher. A member of the research team not involved in scoring observed a random 20% of sessions using the fidelity checklist; mean adherence was 94% (range 88%–100%). Deviations were reviewed and corrected at the monthly meetings.

### 3.4 Evaluation tools and indicators

#### 3.4.1 Theoretical assessment score

A uniformly developed neurological nursing theory test was administered before (pre-test) and after (post-test) the internship. The test covered etiology, clinical manifestations, treatment principles, and nursing key points of common neurological diseases, including multiple-choice (40 points), fill-in-the-blank (20 points), short-answer (30 points), and case analysis (10 points) questions, totaling 100 points.

#### 3.4.2 Clinical practice ability assessment

Assessed via Objective Structured Clinical Examination (OSCE) after the internship. Three stations were established: Nursing Assessment Station (30 points), Skill Operation Station (40 points), and Emergency Management Station (30 points), totaling 100 points. The Nursing Assessment Station required a comprehensive nursing assessment and report for a simulated stroke patient. The Skill Operation Station assessed core nursing procedures like positioning and limb rehabilitation for stroke patients. The Emergency Management Station simulated an emergency scenario to evaluate emergency response and communication skills. Each station lasted 12 min (36 min total per candidate), with a 3-min rotation interval. All scenarios were standardized using scripted case briefs, standardized patients trained over two preparatory sessions, and identical equipment checklists. Six examiners (two per station) were neurology nursing specialists who completed a half-day calibration workshop using anchored scoring rubrics and benchmark video cases before the examination. Assessment criteria were developed by 3 nursing experts, with an inter-rater reliability of 0.85 (intraclass correlation coefficient, ICC, for the total score; station-level ICCs ranged from 0.81 to 0.88). The same examiners and scenarios were used for both groups.

#### 3.4.3 Critical thinking ability assessment

Critical thinking disposition was assessed using the CTDI-CV before and after the internship. This 70-item instrument comprises seven dimensions: Truth-seeking, Open-mindedness, Analyticity, Systematicity, Critical Thinking Self-confidence, Inquisitiveness, and Cognitive Maturity. Items are rated on a 6-point Likert scale, yielding a total score ranging from 70 to 420, with higher scores representing a stronger disposition. Based on the total score, dispositions are categorized as positive (≥280), medium (210–279), or weak (≤209). The inventory was self-administered on paper under supervision and required approximately 20 min to complete. All 86 participants returned fully completed inventories (response rate 100%), so no imputation for missing items was required; the internal consistency (Cronbach’s *α*) of the total scale in this sample was 0.91.

#### 3.4.4 Teaching satisfaction survey

Teaching satisfaction was measured by a self-developed Nursing Clinical Teaching Satisfaction Questionnaire upon completion of the internship. The questionnaire was grounded in the Kirkpatrick reaction level and the CIPP (Context-Input-Process-Product) evaluation framework, from which the five dimensions were derived. The 20-item questionnaire comprises five dimensions: teaching content, teaching methods, teaching effectiveness, instructor performance, and learning gains. Responses are recorded on a 5-point Likert scale for each item, with total scores ranging from 20 to 100; higher scores reflect greater satisfaction. Content validity was established by a panel of five nursing-education experts; the item-level content validity index (I-CVI) ranged from 0.80 to 1.00 and the scale-level CVI (S-CVI/Ave) was 0.88. Reliability was supported by a Cronbach’s *α* of 0.86 and a two-week test–retest reliability of 0.84 (*n* = 20 in a pilot sample). The full questionnaire is provided in the Supplementary material.

### 3.5 Blinding

Owing to the educational nature of the intervention, instructors and students could not be blinded to group allocation. To reduce assessor bias, theory examinations were machine−/blind-scored against a fixed answer key by graders unaware of group allocation; OSCE examiners and the data analyst were also blinded to allocation, and OSCE candidates were identified only by code. Complete blinding of all parties was not feasible, and residual performance and detection bias cannot be excluded.

### 3.6 Data collection

Prior to data collection, the research purpose, methods, and significance were explained in detail to participants, and informed consent was obtained. Trained researchers collected data to ensure authenticity and completeness. Theoretical and OSCE assessments were scored using uniform standards. The CTDI-CV and satisfaction questionnaire were completed independently by students and collected on-site. All 86 distributed questionnaires were returned and valid, yielding a 100% effective response rate.

### 3.7 Statistical analysis

Statistical analyses were performed with SPSS 25.0 (IBM Corp., Armonk, NY, USA). The distribution of continuous variables was examined with the Shapiro–Wilk test and by inspection of Q–Q plots; homogeneity of variance was checked with Levene’s test. Continuous data are presented as mean ± standard deviation (x̄ ± *s*). Within-group (pre- vs. post-test) comparisons used paired-samples t-tests and between-group comparisons used independent-samples *t*-tests. Because some variables in this modest sample did not strictly meet normality assumptions, all primary comparisons were corroborated with non-parametric tests (Wilcoxon signed-rank for within-group and Mann–Whitney U for between-group comparisons); the parametric and non-parametric results were concordant, and exact Mann–Whitney results for the principal outcomes are reported in the table footnotes. Categorical data are expressed as frequency and percentage and were compared using the *χ*^2^ test. To quantify the magnitude of effects, Cohen’s d was calculated for all between-group comparisons (d ≈ 0.2, 0.5, and 0.8 denoting small, medium, and large effects). For the seven CTDI-CV subscales, a Bonferroni-corrected significance threshold of *α* = 0.05/7 = 0.007 was applied to control the family-wise error rate. Two-sided *p* < 0.05 was considered statistically significant for the primary outcomes. Missing data were handled by complete-case analysis; no outcome data were missing.

## 4 Results

### 4.1 Comparison of general characteristics between groups

Baseline demographic and academic characteristics were comparable between groups (Table 1). The control group included 5 males and 38 females (mean age 21.56 ± 0.87 years), while the experimental group comprised 4 males and 39 females (mean age 21.78 ± 0.92 years). No significant differences were observed in educational background (undergraduate/college: control, 22/21; experimental, 23/20). Prior to the internship, the basic nursing theory scores were 79.32 ± 6.45 in the control group and 78.95 ± 6.73 in the experimental group. No statistically significant differences were observed between the two groups in gender distribution, age, educational background or pre-internship theory scores, confirming the baseline equivalence of the groups.

**Table 1:** Comparison of general characteristics between the two groups.

General characteristic	Control group (*n* = 43)	Experimental group (*n* = 43)	*χ*^2^/*t* value	*p*-value
Gender (male/female, n)	5/38	4/39	0.162	0.687
Age (years, x̄ ± s)	21.56 ± 0.87	21.78 ± 0.92	1.053	0.295
Educational background (undergraduate/college, n)	22/21	23/20	0.093	0.761
Pre-internship basic nursing theory score (x̄ ± s)	79.32 ± 6.45	78.95 ± 6.73	0.268	0.789

Continuous variables compared with independent-samples *t*-tests; categorical variables with the *χ*^2^ test. All *p* > 0.05, confirming baseline comparability.

### 4.2 Comparison of teaching effectiveness between groups

#### 4.2.1 Comparison of theoretical assessment scores

The pre- and post-internship theoretical assessment scores are summarized in Table 2. Before the intervention, the mean scores were 79.32 ± 6.45 for the control group and 78.95 ± 6.73 for the experimental group, with no significant intergroup difference. Following the 4-week internship, the control group’s score changed marginally to 78.45 ± 6.72, a non-significant within-group change. In contrast, the experimental group showed a substantial and statistically significant improvement, achieving a mean post-internship score of 87.62 ± 5.38. The between-group comparison of post-internship scores showed significantly higher scores in the experimental group (t = 7.235, *p* < 0.001), with a large effect size (Cohen’s d = 1.51). Educationally, the 9.17-point advantage corresponds to a shift from a “pass” to a “good” performance band on the 100-point examination.

**Table 2:** Comparison of theoretical assessment scores before and after internship between groups (points, x̄ ± s).

Group	*n*	Pre-internship	Post-internship	*t* (within-group)	*p* (within-group)	Cohen’s d (post)
Control group	43	79.32 ± 6.45	78.45 ± 6.72	0.689	0.493	—
Experimental group	43	78.95 ± 6.73	87.62 ± 5.38	6.924	<0.001	1.51

Within-group comparisons by paired *t*-test. Between-group post-internship comparison: *t* = 7.235, *p* ≤ 0.001, Cohen’s d = 1.51 (large). Baseline between-group comparison: *t* = 0.268, *p* = 0.789. Non-parametric corroboration (Mann–Whitney *U*) was concordant (*Z* = −6.05, *p* ≤ 0.001).

#### 4.2.2 Comparison of clinical practice ability

The results of the OSCE, detailed in Table 3, indicated superior clinical performance by the experimental group across all assessed domains. In the Nursing Assessment Station, the experimental group scored 27.68 ± 2.15 points, considerably higher than the control group’s 23.45 ± 3.26 (d = 1.53). Similarly, in the Skill Operation Station, the experimental group achieved 35.76 ± 3.28 points compared to 30.28 ± 4.15 in the control group (d = 1.47). For the Emergency Management Station, scores were 26.92 ± 2.95 and 22.55 ± 3.87 for the experimental and control groups, respectively (d = 1.27). Consequently, the total OSCE score was significantly higher in the experimental group (85.36 ± 4.89) than in the control group (76.28 ± 5.64) (*t* = 8.234, *p* < 0.001; d = 1.72).

**Table 3:** Comparison of OSCE scores between groups (points, x̄ ± s).

Assessment dimension	Control group (*n* = 43)	Experimental group (*n* = 43)	*t* value	*p*-value	Cohen’s d
Nursing Assessment Station	23.45 ± 3.26	27.68 ± 2.15	7.542	<0.001	1.53
Skill operation station	30.28 ± 4.15	35.76 ± 3.28	6.893	<0.001	1.47
Emergency management station	22.55 ± 3.87	26.92 ± 2.95	5.987	<0.001	1.27
Total score	76.28 ± 5.64	85.36 ± 4.89	8.234	<0.001	1.72

Between-group comparisons by independent-samples *t*-test; all *p* ≤ 0.001. Mann–Whitney *U* tests were concordant for every station (all *p* ≤ 0.001). All effect sizes are large (d ≥ 0.8).

#### 4.2.3 Comparison of critical thinking ability

Changes in critical thinking disposition, as measured by the CTDI-CV, are displayed in Table 4, which presents the between-group post-internship comparisons with t-values, *p*-values, and Cohen’s d. At baseline, there were no significant differences between the groups in the total score or any of the seven subscale scores (all *p* > 0.05). After the internship, the experimental group showed higher scores. The CTDI-CV total score was 289.57 ± 18.63 in the experimental group versus 269.63 ± 20.87 in the control group (*t* = 4.67, *p* < 0.001; d = 1.01). Significant between-group differences in post-internship scores were also observed in six subscales: Truth-seeking (42.36 ± 3.89 vs. 39.15 ± 4.36), Open-mindedness (41.58 ± 3.76 vs. 38.72 ± 4.15), Analyticity (41.25 ± 3.96 vs. 37.89 ± 4.28), Systematicity (42.18 ± 3.85 vs. 39.05 ± 4.12), Critical Thinking Self-confidence (41.36 ± 3.98 vs. 38.15 ± 4.36), and Inquisitiveness (43.25 ± 3.87 vs. 40.05 ± 4.25), all remaining significant after Bonferroni correction (all *p* < 0.001; d = 0.72–0.82). However, no statistically significant difference was found in the Cognitive Maturity dimension (39.25 ± 4.15 vs. 37.72 ± 4.42; *p* = 0.102; d = 0.36).

**Table 4:** Between-group comparison of post-internship CTDI-CV scores (points, x̄ ± *s*).

Dimension	Control group (*n* = 43)	Experimental group (*n* = 43)	*t* value	*p*-value	Cohen’s d
Truth-seeking	39.15 ± 4.36	42.36 ± 3.89	3.60	<0.001	0.78
Open-mindedness	38.72 ± 4.15	41.58 ± 3.76	3.35	0.001	0.72
Analyticity	37.89 ± 4.28	41.25 ± 3.96	3.78	<0.001	0.82
Systematicity	39.05 ± 4.12	42.18 ± 3.85	3.64	<0.001	0.79
Critical thinking self-confidence	38.15 ± 4.36	41.36 ± 3.98	3.56	<0.001	0.77
Inquisitiveness	40.05 ± 4.25	43.25 ± 3.87	3.65	<0.001	0.79
Cognitive maturity	37.72 ± 4.42	39.25 ± 4.15	1.65	0.102	0.36
Total score	269.63 ± 20.87	289.57 ± 18.63	4.67	<0.001	1.01

Between-group post-internship comparisons were made using independent-samples *t*-tests. A Bonferroni-corrected threshold (*α* = 0.05/7 = 0.007) was applied to the seven subscales: six remained significant; Cognitive Maturity did not (*p* = 0.102; small effect, d = 0.36). At baseline, no between-group difference reached significance for the total or any subscale (all *p* ≥ 0.05). Mann–Whitney *U* tests were concordant.

#### 4.2.4 Comparison of teaching satisfaction

**Figure 2:**
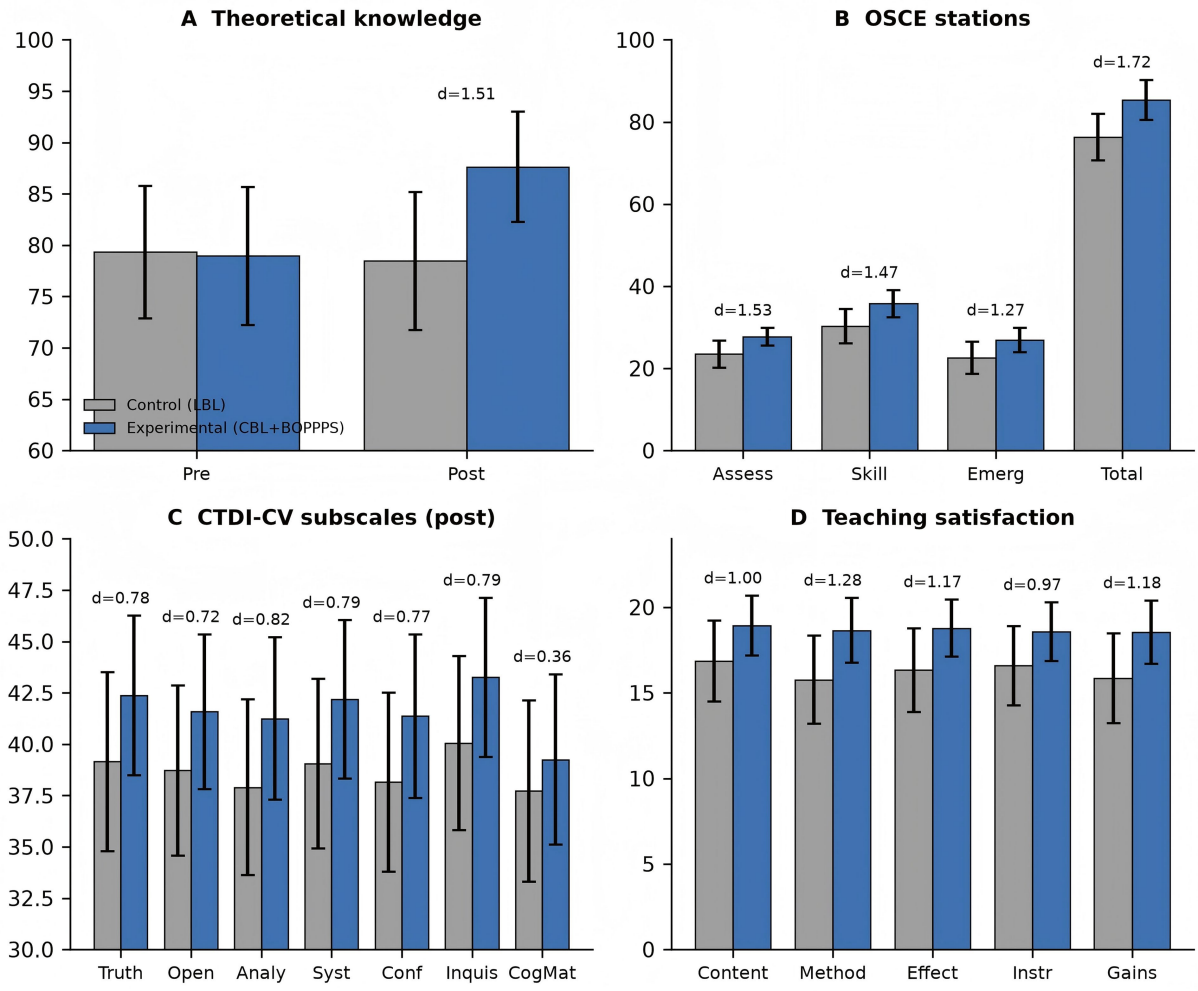
Between-group outcomes with Cohen’s d effect sizes (mean ± SD). **(A)** Pre- and post-intervention theoretical knowledge scores. **(B)** Individual stations and total scores of the OSCE. **(C)** Post-intervention subscale scores of the California Critical Thinking Disposition Inventory (CTDI-CV). **(D)** Subscale scores of the teaching satisfaction questionnaire. Pre = pre-intervention baseline assessment; Post = post-intervention assessment; Assess = assessment; Skill = clinical skill; Emerg = emergency care; Truth = truth-seeking; Open = open-mindedness; Analy = analyticity; Syst = systematicity; Conf = confidence in reasoning; Inquis = inquisitiveness; CogMat = cognitive maturity; Content = teaching content; Method = teaching method; Effect = teaching effect; Instr = instructor performance; Gains = personal gains.

Teaching satisfaction outcomes are presented in Table 5. The experimental group reported significantly higher satisfaction levels across all five dimensions surveyed. For Teaching Content, the score was 18.92 ± 1.75 in the experimental group versus 16.85 ± 2.36 in the control group. Regarding Teaching Methods, scores were 18.65 ± 1.89 and 15.76 ± 2.58, respectively. Satisfaction with Teaching Effectiveness was also higher in the experimental group (18.78 ± 1.67 vs. 16.32 ± 2.45). Similarly, Instructor Performance received a score of 18.56 ± 1.72 in the experimental group compared to 16.58 ± 2.32 in the control group. Finally, perceived Learning Gains were rated at 18.54 ± 1.85 in the experimental group and 15.85 ± 2.63 in the control group. The overall total satisfaction score was significantly higher for the experimental group (92.45 ± 3.67) than for the control group (81.36 ± 5.28) (*t* = 11.234, *p* < 0.001; d = 2.44). The effect sizes for all primary outcomes are visually summarized in Figure 2.

**Table 5:** Comparison of teaching satisfaction between groups (points, x̄ ± *s*).

Dimension	Control group (*n* = 43)	Experimental group (*n* = 43)	*t* value	*p*-value	Cohen’s d
Teaching content	16.85 ± 2.36	18.92 ± 1.75	4.893	<0.001	1.00
Teaching methods	15.76 ± 2.58	18.65 ± 1.89	6.234	<0.001	1.28
Teaching effectiveness	16.32 ± 2.45	18.78 ± 1.67	5.765	<0.001	1.17
Instructor performance	16.58 ± 2.32	18.56 ± 1.72	4.987	<0.001	0.97
Learning gains	15.85 ± 2.63	18.54 ± 1.85	5.678	<0.001	1.18
Total score	81.36 ± 5.28	92.45 ± 3.67	11.234	<0.001	2.44

Between-group comparisons by independent-samples *t*-test; all *p* ≤ 0.001. Mann–Whitney *U* tests were concordant. Effect sizes range from large to very large (d = 0.97–2.44).

## 5 Discussion

With the intensifying trend of population aging, the incidence of neurological diseases is rising annually, placing higher demands on the professional competence and practical skills of neurological nursing personnel. The highly specialized nature of neurological nursing entails significant challenges, requiring interns to develop an integrated skill set encompassing theoretical knowledge, clinical proficiency, critical thinking, and emergency response capabilities. As a critical link between nursing theory and practice, the quality of clinical practice education directly influences the effectiveness of nursing personnel training. This study was designed to answer three research questions: whether the integrated CBL–BOPPPS model yields superior gains in (1) theoretical knowledge and OSCE-assessed clinical skills (RQ1), (2) critical thinking disposition and its sub-dimensions (RQ2), and (3) teaching satisfaction (RQ3), compared with traditional LBL. The following discussion addresses each question sequentially, interprets the findings against prior evidence, and discusses their implications for neurological nursing education.

The traditional clinical teaching model is teacher-centered, with students passively receiving knowledge and lacking opportunities for active thinking and practical application. Within this model, students lack practical training in independent analysis and problem-solving, leading to a disconnect between theory and practice, suboptimal cultivation of clinical reasoning and practical skills, and room for improvement in both teaching quality and student satisfaction (21, 22). Therefore, exploring scientific and efficient clinical teaching models has become an important direction for reform in neurological nursing clinical practice education.

Regarding the rationale for integration, CBL and BOPPPS are complementary rather than redundant. BOPPPS contributes an explicit, closed-loop structure—objectives, diagnostic pre-assessment, participatory activity, post-assessment, and feedback—whereas CBL supplies the authentic clinical content that gives each phase meaning. Embedding one longitudinal neurological case across all six phases means that diagnostic pre-assessment targets case-specific gaps, participatory learning operationalizes the case, and post-assessment verifies case-based decisions, producing a coherence that neither model achieves alone (7, 18, 19). This theoretical complementarity is the mechanism we propose for the observed gains.

In response to RQ1, the integrated CBL–BOPPPS model is indeed associated with significantly greater gains in both theoretical knowledge and OSCE-assessed clinical skills compared to traditional LBL. Specifically, the experimental group outperformed the control group by a clinically meaningful margin in theoretical scores (87.62 ± 5.38 vs.78.45 ± 6.72, d = 1.51), representing an educational shift from a borderline-pass to a solidly “good” performance band. These findings align with and extend prior research across various medical specialties. For instance, Chen et al. (7) and Gao et al. (18) demonstrated that integrating CBL within the BOPPPS framework significantly outperformed LBL in theoretical assessments among ophthalmology and oral surgery students, respectively. More recently, a multi-cohort quasi-experimental study among undergraduate nursing students found that the BOPPPS-CBL model significantly enhanced knowledge acquisition in healthcare-associated infection education (23). Furthermore, a comprehensive review by Yao et al. (11) confirmed that CBL interventions effectively bridge the gap between abstract nursing theories and clinical contexts. However, our observed effect size for theoretical knowledge (d = 1.51) is notably robust. This pronounced gain may be attributed to the unique characteristics of neurological nursing, which demands the rapid memorization and application of complex, time-sensitive pathways (e.g., stroke thrombolysis windows, seizure management protocols) (24, 25). The explicit “Objective” setting and the closed-loop “Pre-/Post-assessment” phases of BOPPPS likely provided the necessary cognitive scaffolding, ensuring that students not only understood the case but also rigorously internalized the high-stakes theoretical concepts required for neurological care.

Also addressing RQ1, the OSCE results indicate that the integrated model was associated with substantially enhanced clinical practical abilities (85.36 ± 4.89 vs. 76.28 ± 5.64; d = 1.72). This corroborates the efficacy of structured, case-anchored learning seen in other disciplines (30, 31). For example, Gao et al. (18) reported enhanced surgical skills in oral and maxillofacial surgery using a similar integrated model, and Xiao et al. (20) highlighted the benefits of a longitudinal “One Case to the End” approach in pediatric nursing. Beyond these settings, similar benefits have been documented in emergency medicine training (11) and nursing skill acquisition (13), underscoring the model’s generalizability across health professions. Building upon these studies, our intervention introduces a critical dimension specific to neurology: emergency response in time-critical scenarios. Neurological emergencies, such as acute ischemic stroke or status epilepticus, require not only flawless technical execution but also rapid clinical judgment under pressure. By embedding emergency simulations within the BOPPPS “Participatory Learning” and “Post-assessment” phases, and utilizing a longitudinal case that evolves with the patient’s condition changes, students were forced to continuously adapt their care plans in real-time (33). This dynamic, high-acuity simulation likely explains the pronounced gains observed specifically in our Emergency Management Station (d = 1.27). Ultimately, the integration ensures that the structural rigor of BOPPPS prevents the “discussion drift” sometimes seen in pure CBL, while the authentic neurological cases prevent the BOPPPS model from becoming a mere mechanical checklist, thereby optimally narrowing the gap between theoretical knowledge and life-saving clinical actions.

In response to RQ2, the integrated CBL–BOPPPS model is associated with significantly improved critical thinking dispositions, particularly in six of the seven CTDI-CV dimensions. The experimental group achieved a higher total CTDI-CV score (289.57 ± 18.63 vs. 269.63 ± 20.87; d = 1.01) and showed significant gains in Truth-seeking, Open-mindedness, Analyticity, Systematicity, Self-confidence, and Inquisitiveness. The meta-analysis by Xiang et al. (12) corroborates our findings, demonstrating that CBL interventions significantly enhance critical thinking dispositions among Chinese nursing students. Moreover, our results resonate with the work of Zainal et al. and Dang et al. (26, 34), who highlighted that critical thinking is not only a fundamental prerequisite for effective clinical decision-making but also plays a decisive role in medical ethical decision-making in complex clinical practice. The gains in Analyticity and Systematicity are particularly crucial for neurological nursing, where managing variable and multi-system diseases requires highly structured and analytical reasoning. The medium-to-large effect sizes (d = 0.72–0.82) confirm that these improvements are practically as well as statistically meaningful. Mechanistically, the CBL component encourages the collision of diverse viewpoints during group discussions, stimulating deep cognitive processing (27), while the BOPPPS “Summary” and “Post-assessment” phases enforce structured reflection, thereby reinforcing self-confidence and inquisitiveness (12).

Notably, concerning the Cognitive Maturity dimension, the between-group difference did not reach statistical significance (39.25 ± 4.15 vs. 37.72 ± 4.42; *p* = 0.102; d = 0.36). Rather than viewing this as a limitation of the integrated model, this null finding offers a critical insight into the developmental trajectory of critical thinking and provides a clear direction for future curriculum design. Cognitive maturity—the disposition to recognize the complexity of problems and to make judgments under uncertainty—is conceptualized in recent literature not as a short-term malleable state, but as a deeply ingrained, slower-changing trait (28). This interpretation is consistent with findings from a quasi-experimental study by Amouzeshi et al. (29), which observed similarly limited changes in cognitive maturity following a 4-week intervention, suggesting that this dimension may require extended reinforcement beyond a single internship block. Developing this trait requires prolonged, repeated exposure to authentic clinical ambiguities and diagnostic uncertainties, which a 4-week (8-session) internship cycle is likely insufficient to achieve. While the integrated model effectively sharpened more active and immediate thinking skills (e.g., analyticity and inquisitiveness), altering foundational cognitive maturity requires a longer longitudinal trajectory. Additionally, a post-hoc power analysis indicated that detecting this small effect size (d = 0.36) would require approximately 98 participants per group, suggesting our modest sample was under-powered for this specific subscale. For future optimization, we recommend extending the intervention duration and incorporating targeted meta-cognitive scaffolds, such as reflective journaling and structured debriefing focused explicitly on diagnostic uncertainty, to better cultivate cognitive maturity in neurological nursing students.

In response to RQ3, students in the experimental group reported markedly higher teaching satisfaction than their counterparts in the control group across all five surveyed dimensions, with a substantially higher total score (92.45 ± 3.67 vs. 81.36 ± 5.28; d = 2.44). This pronounced difference is corroborated by a meta-analysis (17), which confirmed that the BOPPPS model significantly enhances course satisfaction in nursing education by providing clear learning trajectories and continuous formative feedback. Similar findings have been reported in recent BOPPPS-based nursing courses (15, 16), where students consistently preferred the structured yet interactive environment. Beyond the inherent structural advantages of the BOPPPS framework, the exceptionally high satisfaction observed in our study may be uniquely attributable to the implementation of a longitudinal “One Case to the End” approach (20). Unlike traditional LBL or fragmented case presentations that depict diseases as isolated snapshots, our intervention utilized a single neurological case that evolved dynamically across the six BOPPPS phases. This longitudinal design minimizes extraneous cognitive load by allowing students to trace the complete clinical trajectory of a disorder—from initial presentation and acute intervention to ongoing management—while maintaining narrative coherence and clinical relevance. Furthermore, the Participatory Learning and Post-assessment phases transform students from passive recipients of information into active clinical decision-makers. This shift bridges the gap between abstract theory and bedside practice and fulfills students’ psychological needs for autonomy and competence, which are critical drivers of intrinsic learning satisfaction.

From a theoretical perspective, this study extends constructivist and situated learning theories into the highly complex, time-sensitive context of neurological nursing, providing empirical validation for the “structure–content” complementary framework proposed in the literature. Furthermore, our nuanced finding regarding the CTDI-CV subscales contributes to the theoretical understanding of critical thinking development. By distinguishing between short-term malleable dispositions (e.g., analyticity, inquisitiveness) and foundational traits (e.g., cognitive maturity) that require prolonged exposure to clinical ambiguity, this study refines the theoretical trajectory of critical thinking cultivation in nursing education. Practically, the findings offer actionable insights for clinical nursing educators and curriculum designers. The integrated “One Case to the End” protocol provides a standardized, highly replicable teaching blueprint for specialized wards—one that leverages the longitudinal case structure to reduce cognitive load and foster systematic clinical reasoning, as discussed above. For nursing education managers, this study underscores the necessity of transitioning from fragmented, static case lectures toward coherent, dynamic case trajectories in clinical rotation curricula. Finally, consistent with our discussion of cognitive maturity above, educators should recognize that certain foundational dispositions may require longitudinal curricular reinforcement beyond a single rotation, and we therefore encourage the adoption of sustained meta-cognitive strategies to foster students’ tolerance for clinical ambiguity.

This study has limitations. Foremost, the non-randomized, quasi-experimental design with allocation by internship batch limits causal inference and is susceptible to selection and temporal confounding; the findings should therefore be interpreted as associations rather than definitive causal effects. It is also a single-center study with a sample size of only 86 students, which may limit the generalizability of the results. Although outcome assessors were blinded, instructors and students were not, leaving residual performance and detection bias. The use of a self-developed satisfaction questionnaire may introduce subjective bias despite acceptable validity evidence. The evaluation period was relatively short, assessing only immediate post-internship effects without tracking long-term retention or transfer to practice. Finally, although primary results were robust to non-parametric corroboration and Bonferroni correction, the study was not powered for small subscale effects. In the future, we will conduct adequately powered, randomized, multi-center studies with longitudinal follow-up, optimize the teaching protocol for slower-changing dimensions such as cognitive maturity, and employ more objective evaluation tools to strengthen causal inference.

## 6 Conclusion

In this single-centre quasi-experimental study, the integrated CBL and BOPPPS model, which embeds CBL within each phase of the BOPPPS framework, was associated with improved mastery of theoretical knowledge, clinical practical ability, and critical thinking disposition, and with higher learning satisfaction among neurological nursing interns. Because the design was non-randomized and single-centre, these results should be regarded as promising but preliminary. Future directions include adequately powered, randomized, multi-centre trials with long-term follow-up, targeted strategies for slower-changing dispositions such as cognitive maturity, and the use of more objective outcome measures, so as to confirm effectiveness before broad adoption in specialized nursing education.

## Data Availability

The raw data supporting the conclusions of this article will be made available by the authors, without undue reservation.
