# Supplementary material for: The effectiveness of an integrated CBL and BOPPPS model in neurological nursing clinical teaching: a comparative study
Source: Front Med (Lausanne). 2026 Jul 29;13:1789025. doi: 10.3389/fmed.2026.1789025 (PMC13463138; doi:10.3389/fmed.2026.1789025)
Supplement: Supplementary file 1 [file Data_Sheet_1.DOCX]

**Supplementary Material**

**Title:** The Effectiveness of an Integrated CBL and BOPPPS Model in Neurological Nursing Clinical Teaching: A Comparative Study

**Running title:** Integrated CBL–BOPPPS Model in Neurological Nursing

**Authors:** Peijun Qian*, Juan Su, Yaoyao Xu

**Affiliation:**

Department of Neurology, The Second Affiliated Hospital of Anhui Medical University, Hefei City, Anhui Province, 230601, China

***Corresponding author:**

Peijun Qian

Department of Neurology, The Second Affiliated Hospital of Anhui Medical University, No. 678, Furong Road, Hefei City, Anhui Province, 230601, China

Tel./Fax: +86-551-6386-9420;

Email: pjqian0658@163.com

**Appendix S1. Nursing Clinical Teaching Satisfaction Questionnaire (self-developed)**

Instructions: Please rate your agreement with each statement about your neurology internship teaching on a 5-point Likert scale: 1 = strongly disagree, 2 = disagree, 3 = neutral, 4 = agree, 5 = strongly agree. The questionnaire contains 20 items across five dimensions (4 items each); total score 20–100. Content validity: I-CVI 0.80–1.00, S-CVI/Ave 0.88 (five experts); Cronbach's α = 0.86; two-week test-retest = 0.84. Theoretical basis: Kirkpatrick (reaction) and CIPP evaluation frameworks.

**Dimension 1 — Teaching Content**

1. The teaching content was relevant to clinical neurological nursing practice. (1 2 3 4 5)

2. The content was appropriately challenging for my level. (1 2 3 4 5)

3. The content was well organized and logically sequenced. (1 2 3 4 5)

4. The case material reflected authentic clinical situations. (1 2 3 4 5)

**Dimension 2 — Teaching Methods**

5. The teaching methods encouraged my active participation. (1 2 3 4 5)

6. Group discussion and role-play helped me learn. (1 2 3 4 5)

7. The methods stimulated my interest in neurological nursing. (1 2 3 4 5)

8. The balance between guidance and independent work was appropriate. (1 2 3 4 5)

**Dimension 3 — Teaching Effectiveness**

9. The teaching improved my understanding of neurological nursing theory. (1 2 3 4 5)

10. The teaching improved my clinical reasoning ability. (1 2 3 4 5)

11. The teaching improved my practical nursing skills. (1 2 3 4 5)

12. The teaching improved my confidence in handling emergencies. (1 2 3 4 5)

**Dimension 4 — Instructor Performance**

13. Instructors were well prepared for each session. (1 2 3 4 5)

14. Instructors provided timely and constructive feedback. (1 2 3 4 5)

15. Instructors created a supportive learning atmosphere. (1 2 3 4 5)

16. Instructors responded clearly to my questions. (1 2 3 4 5)

**Dimension 5 — Learning Gains**

17. I can apply what I learned to real patient care. (1 2 3 4 5)

18. My problem-solving ability improved during the internship. (1 2 3 4 5)

19. My motivation for continued learning increased. (1 2 3 4 5)

20. Overall, this internship met my learning expectations. (1 2 3 4 5)

**Appendix S2. Critical Thinking Disposition Inventory – Chinese Version (CTDI-CV)**

The CTDI-CV is a validated, copyrighted 70-item instrument (seven dimensions; 6-point Likert; total 70–420) and is therefore cited rather than reproduced here. In this study it was self-administered on paper under supervision (~20 minutes), with a 100% response rate, no missing items, and sample Cronbach's α = 0.91.
